# Epistaxis Prevention, Treatment, and Future Perspectives for Hereditary Hemorrhagic Telangiectasia

**DOI:** 10.3390/jcm14217724

**Published:** 2025-10-30

**Authors:** Anthony Ficany, Marta Del Alamo, Carmelo Bernabeu, Claire L. Shovlin, Elisa Rossi

**Affiliations:** 1 INSERM, U1144 Optimisation Thérapeutique en Neuropharmacologie (OPTeN), Faculty of Pharmacy, Université Paris-Cité, 75006 Paris, France; anthony.ficany@u-paris.fr; 2 ECRIN–European Clinical Research Infrastructure Network, 75014 Paris, France; marta.delalamo@ecrin.org; 3 Department of Biomedicine, Centro de Investigaciones Biológicas “Margarita Salas”, Consejo Superior de Investigaciones Científicas (CSIC), 28040 Madrid, Spain; bernabeu.c@cib.csic.es; 4 National Heart and Lung Institute, Imperial College London, London W12 0NN, UK; c.shovlin@imperial.ac.uk; 5 Specialist Medicine, Imperial College Healthcare NHS Trust, London W12 0HS, UK

**Keywords:** HHT, epistaxis, clinical trials, new therapeutic approaches

## Abstract

Hereditary Hemorrhagic Telangiectasia (HHT), also known as Osler-Weber-Rendu syndrome, is a vascular disorder with a global prevalence ranging from 1:5000 to 1:8000. It most commonly manifests through nosebleeds, which can be frequent and severe, exposing patients to major iron losses, anemia, and considerable physical and emotional distress. To date, no drug has received the FDA or EMA approval for preventing or treating HHT associated epistaxis, limiting access to therapies and intensifying the burden on patients and clinicians. Based on peer-reviewed evidence, the Second International HHT Guidelines provided a stepwise approach to help physicians manage HHT-related epistaxis highlighting the role of anti-fibrinolytic and systemic antiangiogenic drugs. However, experience from clinical practice and trials indicates marked variability in patient responses, and none of the recommended approaches has demonstrated sufficient placebo-controlled efficacy to gain regulatory approval. Striking insights in HHT physiopathology shed light on complex dysregulated signaling pathways with a triggering role not only by angiogenesis as widely recognized, but also by inflammation, injury and other stimuli, pointing to novel therapeutic targets. This review outlines current recommendations for preventing and managing nosebleeds in HHT patients, highlights the latest insights into the development of telangiectasic lesions, and discusses potential therapeutic treatments currently under clinical investigation.

## 1. Introduction

Hereditary hemorrhagic telangiectasia (HHT), also known as Osler–Weber–Rendu syndrome, is an autosomal dominant disorder characterized by vascular malformations including small telangiectases that bleed frequently in the nose and gastrointestinal tract, and large arteriovenous malformations (AVMs) where rarer consequences may also include hemorrhage [1]. The diagnosis of HHT is established when a suspected patient bears a pathogenic variant (‘mutation’) in *ENG*, *ACVRL1*, *SMAD4*, or *GDF2* or presents with at least three of the four Curaçao criteria [2,3]. Epidemiological studies estimated the prevalence of HHT to be between 1:5000 and 1:8000, affecting approximately 85,000 individuals in Europe [1,4]. Notably, these figures are subject to substantial under-ascertainment due to lack of classical features in many patients with genetically confirmed HHT [5]. For instance, the Curaçao criteria are less reliable in the pediatric population, since nosebleeds are common in all children, and many children with confirmed HHT will not develop telangiectasia until later in life [6]. In well-defined populations subject to intensive scrutiny, such as the Netherlands Antilles (Curaçao, Bonaire) or the Jura Valley in France, HHT prevalence reached 1 in 1331 individuals and 1 in 2300 individuals, respectively [7,8]. Further socioeconomic factors contributing to underdiagnosis are recognized [9] and a prevalence 2 to 12 times higher than current estimates has been recently proposed based on allelic frequencies [10].

### 1.1. Genetics and Clinical Manifestations

**Figure 1:**
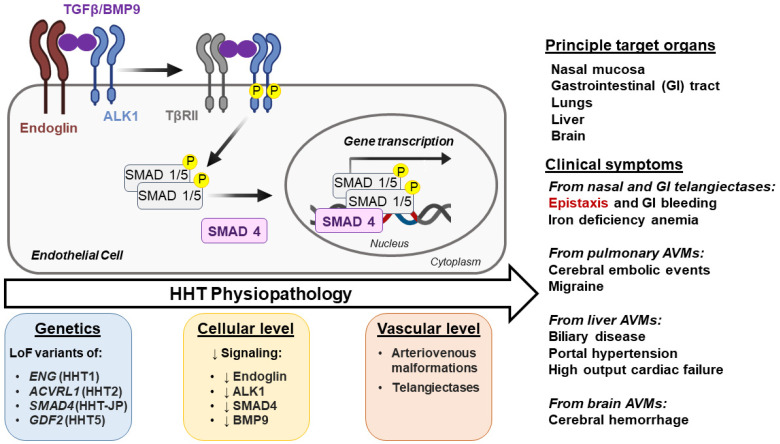
Physiopathology of Hereditary hemorrhagic telangiectasia. The arrow ‘HHT Physiopathology’ indicates progression from basic genetic defect to clinical phenotype. TGF-β/BMP9 Signaling: TGF-β/BMP9 binds to the type I TGFβ receptor ALK1 and to type II TGF-β receptors (TβRII). The ligand binding, facilitated by ALK1 co-receptor endoglin, leads to the formation of a tetrameric receptor complex, allowing TβRII to phosphorylate the cytoplasmic regulatory domain of ALK1, stimulating its own kinase activity. Phosphorylated ALK1 transduces the signal by phosphorylating cytoplasmic Mothers against decapentaplegic homolog (MADH or SMAD) 1/5 proteins. Phosphorylated SMADs bind to the common partner SMAD4, and the resulting complex translocates to the nucleus, binds DNA, and regulates the expression of genes involved in vascular development. Defects in TGFβ/BMP9 signaling at specific times lead to abnormal vessel development and have been used to define hereditary hemorrhagic telangiectasia (HHT) subtypes. Loss of function (LoF) mutations of endoglin (*ENG*), ALK1 (*ACVRL1*), BMP9 (*GDF2*), and SMAD4 (*MADH4*) cause HHT1, HHT2, HHT5, and JP/HHT, respectively. Phosphorylated proteins are denoted by the letter “P” in yellow background. Downward arrows (↓) indicate a decrease.

HHT is inherited as an autosomal dominant trait. All causal genes encode a protein mediating bone morphogenetic protein (BMP)/transforming growth factor β (TGF-β) signaling, and all pathogenic variants are loss of function mutations, including deletions, insertions, missense changes, or splice site alterations [11,12]. In the majority of cases, HHT is due to a heterozygous loss of function variant in *ENG* (HHT1, OMIM # 187300) or *ACVRL1* (HHT2, OMIM # 600376). *ENG* and *ACVRL1* encode the endoglin and ALK1 (activin receptor-like kinase 1) proteins, respectively. Notably, both of these are transmembrane receptors for BMP/TGF-β ligands that share common signaling functions, especially in endothelial cells (Figure 1). Loss-of-function variants in *SMAD4* (previously *MADH4*), which encodes a downstream effector, cause an HHT-juvenile polyposis overlap syndrome (JP-HHT, OMIM # 175050). Additionally, variants in *GDF2* which encodes the high affinity endoglin and ALK1-ligand BMP9 have been shown to cause HHT meeting the Curaçao criteria (OMIM # 615506), though this is rare [13]. Recent whole genome sequencing studies have formally ruled out a previous candidate locus on chromosome 5 (HHT3) [14], and it no longer has an OMIM entry. A candidate locus HHT4 on chromosome 7 still has an independent OMIM entry (OMIM # 610655), but no HHT causal gene has been found since its identification by linkage analysis 20 years ago [15].

As noted above, HHT displays varying penetrance and expression, meaning manifestations vary between affected individuals from silent to more severe clinical consequences according to tissues and organs affected. Patients with HHT typically present with nosebleeds, arteriovenous malformations (AVMs)-induced complications, incidental detection of AVMs, or through family screening programs where genetic testing is identifying pauci-symptomatic patients [16]. Both AVMs and telangiectases are vascular abnormalities characterized by the loss of the capillary bed, leading to abnormal direct connections between arteries and veins. In contrast to proliferative AVM syndromes, where facial and cutaneous AVMs predominate the clinical spectrum [17,18], the HHT AVMs are in internal organs (primarily the lungs, liver, and brain) and are relatively stable structures. The telangiectatic small vessels visible on mucocutaneous surfaces, including the skin, gastrointestinal (GI) tract, and nasal cavity [19], are more dynamic, becoming more frequent with age, with individual lesions growing, destabilizing, bleeding, and regressing, often in that order [1]. The usually asymptomatic AVMs can lead to serious complications such as stroke and brain abscess (from pulmonary AVMs), hemorrhage (from cerebral and rarely pulmonary AVMs), or high-output cardiac failure and portal hypertension (from hepatic AVMs). Notably, pulmonary and cerebral AVMs are more frequently associated with HHT1, while hepatic AVMs are more common in HHT2. In addition, gastrointestinal bleeding from GI telangiectases may occur. Yet, the most common symptom across all HHT types is recurrent epistaxis, primarily caused by breaches in the fragile walls of telangiectatic lesions in the nasal mucosa [20]. Genetics is part of the reason why epistaxis varies so widely, with HHT mutational subtypes and variants in other genes expected to be important in future HHT care, as addressed further in Section 4.

### 1.2. Burden of HHT-Induced Epistaxis

Over 90% of HHT patients suffer from nosebleeds at some point in their lives, and unlike the common patterns of nosebleeds that affect at least one in two children, for HHT families, nosebleeds often persist or develop in adult life when they commonly become more severe and/or frequent. Patterns are highly variable and include infrequent or short duration bleeds. However, the majority of people with HHT bleed sufficiently enough to need extra iron to supplement their nosebleed losses, with smaller proportions needing regular intravenous iron and blood transfusions for substantial nosebleed losses that can occur many times a day and/or lasting several hours, leading to hemodynamic instability and collapse.

**Figure 2:**
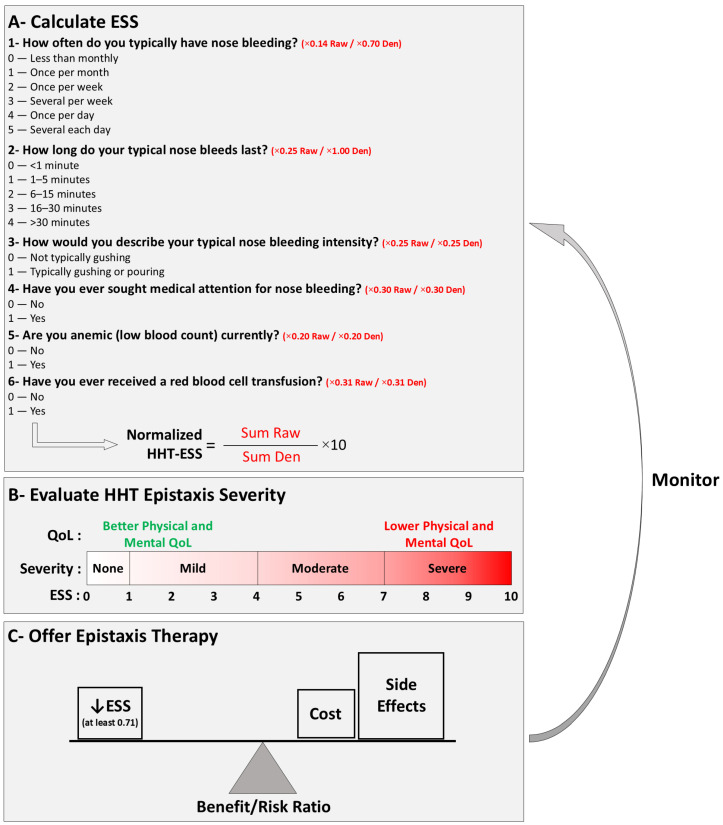
Benefit/Risk Ratio-based approach guided by ESS. (**A**) The epistaxis severity can be quantified by the ESS: For calculation, the patient answers six questions. The number of the response is multiplied (**×**) by the respective coefficient, and the resulting figure gives the raw Epistaxis Severity Score. For normalization, the raw score is divided by the maximum possible score [2,22], then multiplied by 10 [22]. Automated calculation is available online at https://curehht.org/understanding-hht/diagnosis-treatment/nosebleed-severity-score/ (accessed on 25 October 2024) (**B**) Evaluation of Epistaxis Severity. The ESS ranging on a scale from 0 to 10 is inversely correlated with patients’ physical and mental Quality of Life (QoL) [23]. (**C**) Treatment according to Benefit/Risk Ratio. HHT-related epistaxis management involves a wide range of pharmacological and non-pharmacological interventions. However, most efficient therapies are expensive and/or may induce important side effects. The ESS can be a helpful tool to determine a treatment regimen with an optimal benefit/risk ratio (e.g., in patients presenting with a high ESS, the anticipated improvement in quality of life is likely to outweigh the potential side effects and financial burden of therapy). Upon therapy initiation, the ESS can serve as a monitoring tool. The minimum important difference was defined as 0.71 for clinical trials purposes [33], this also implies that treatment should be ceased when it fails to reduce (↓) the ESS by at least 0.71. ESS, Epistaxis Severity Score.

Epistaxis can occur spontaneously; be triggered by minor trauma such as blowing the nose, sneezing, or bending over; by activities or drugs; and by emotional stress, significantly affecting the patients’ Quality of Life (QoL) [21]. The Epistaxis Severity Score (ESS) was developed and approved as a tool to assess the severity of epistaxis based on six factors (frequency, duration, intensity, need for medical attention, anemia, and the need for transfusion) (Figure 2) [22]. Patients with severe nosebleeds have been shown to have a higher ESS and lower QoL than patients with less severe nosebleeds/lower ESS [23]. In addition to consequences from anemia and its treatments, patients report that frequent nosebleeds, and associated anxiety and embarrassment, can make it difficult to perform daily activities, engage socially, and excel academically or professionally [21].

There are additional problems for subgroups of patients with HHT. First, those with iron deficiency as a result of bleeding are at a greater risk of developing pathologies that require blood thinning agents [2] which may further aggravate their bleeds [24]. The most common situations requiring anticoagulation in HHT are venous thromboemboli and atrial fibrillation, both of which are more common if iron deficiency has developed [25,26]. The most common situations requiring antiplatelets in HHT are ischemic strokes and, less commonly, myocardial infarctions due to paradoxical emboli through PAVMs. While embolization and surgery can obliterate many PAVMs, large proportions of treated patients are left with residual PAVMs [27,28]. Ischemic strokes were also shown to be associated with lower serum iron levels [29,30]. Separately, while treatment of anemia and iron deficiency is essential (and may improve nosebleeds directly [31]), there is a small group of HHT patients where iron treatments can make nosebleeds worse [31,32], so judicious care is needed to find optimal regimes.

Together with nosebleed-induced severe anemia that may result in weekly requirements for blood transfusions or intravenous iron infusions, it is clear that the effective treatment of epistaxis plays a pivotal role in the management of HHT (Figure 2).

In the absence of regulatory agency-approved therapies for HHT-related epistaxis management, the Second International HHT Guidelines [2] and a recent International Consensus Report for bleeding in HHT [34], both issued by experts in the field, provide some evidence-based recommendations for treating HHT-related epistaxis [2]. However, there was no attention paid to the prevention of nosebleeds, while many patients suffer from refractory epistaxis that fails to respond to available pharmacological treatments or face severe side effects from proposed therapies. Recent advances in the understanding of HHT physiopathology have revealed novel molecular targets, leading to the development of promising drugs currently in clinical trials. This review outlines the current recommendations for managing nosebleeds in HHT patients and offers a comprehensive analysis of potential therapeutic options under investigation, guided by the latest insights into the underlying mechanisms of HHT-related epistaxis.

### 1.3. Prevention of Nosebleeds

It is unfortunate that it is commonly believed that HHT nosebleed progression is inevitable with age. This and other misconceptions have been recently highlighted by a striking report by senior otorhinological specialists [35]. It is our hope that educational elements will focus on targeting the modifiable triggers of nosebleeds. Among them are the anxiety about nosebleeds and the uncertainty regarding the health prognosis of the patients [21]; personalized approaches to iron requirements [36] and treatments; better emphasis on the variability of nosebleeds, as highlighted by [35]; and better understanding of why some people with HHT have few or no nosebleeds [5]. The following sections of the article focus on the treatment of nosebleeds once these are present.

## 2. Treatment of HHT-Related Epistaxis

In accordance with the Second International HHT Guidelines, the primary management of HHT-related epistaxis involves a variety of topical and systemic approaches aimed at preventing the occurrence and reducing the duration of the event [2]. The panel, comprising clinical and genetic experts in HHT, classifies various interventions in a stepwise-recommended approach. Moisturizing topical therapies are advised for the initial management of nosebleeds. A second line consisting of tranexamic acid (TA) and/or local ablative therapies is advised upon failure of controlling epistaxis. Systemic antiangiogenic agents including bevacizumab are offered as a third-line pharmacological treatment. Considering the risks and benefits of antiangiogenic medications or, in case of refractory epistaxis, alternative non-pharmacological procedures including septodermoplasty or nasal closure can be proposed to prevent bleeding. Notably, the management of acute epistaxis requiring medical intervention includes nasal packing with a product likely to cause rebleeding after removal [2]. Clinical evidence supporting the use of those pharmacological treatments arises from both case report studies and interventional clinical trials [37]. In this comprehensive review, we used the WHO International Clinical Trials Registry Platform (ICTRP) to make an extensive geographical coverage of all interventional clinical trials registered in the Primary Registries of the ICTRP Network and partner registries (including clinicaltrials.gov) testing pharmacological treatments of HHT-related epistaxis. The investigation spanned 52 interventional clinical trials, of which 22 have published the results in peer-reviewed journals (Table 1).

### 2.1. Topical Moisturizing Agents

Humidifying the nasal mucosa using topical agents is a non-invasive approach recommended as the first-line therapy for all HHT patients [2]. Endonasal crusting and airflow can desiccate and damage the nasal epithelium, destabilizing nasal telangiectases. Therefore, moisturizing the nasal mucosa using topical agents and/or an air humidifier should reduce cracking and crusting, limiting bleeding from fragile telangiectasia. These agents include over-the-counter saline sprays, gels, and ointments. For example, topical saline used twice daily significantly decreased the ESS after 12 weeks of treatment in a randomized controlled trial [43]. However, moisturizing the nasal mucosa alone is not effective for many patients, and some report that the application procedure triggers nosebleeds.

### 2.2. Tranexamic Acid

TA is a synthetic antifibrinolytic that inhibits the degradation of fibrin clots by competitively inhibiting the activation of plasminogen to plasmin. At high concentrations, it also non-competitively blocks plasmin [60]. In the ATERO study, a European crossover-controlled trial, TA (1.5 g twice daily) significantly decreased monthly bleeding time in HHT patients without affecting the number of epistaxis per month [59]. Another crossover trial showed that TA (1 g three times daily) improved the ESS without any positive effect on the hemoglobin levels of the patients [58]. In the NOSE study, topical application of TA twice daily showed no significant difference with placebo in terms of improvement of the ESS and hemoglobin level [43]. Based on these results, experts recommend the use of oral TA as an option for managing epistaxis that does not respond to moisturizing topical therapies alone [2]. However, daily administration of TA may require clinical monitoring due to potential adverse reactions, as it can cause ocular defects [61,62], hypersensitivity reactions [63], seizures/myoclonus [64], and thromboembolic events due to fibrinolysis inhibition [65]. Additionally, it may cause dizziness, impairing physical or mental abilities, due to central nervous system effects. Furthermore, 50% of patients receiving oral TA report headaches [66]. Importantly, for patients with kidney impairment, pharmacokinetic studies showed that TA is eliminated at 95% by urinary excretion as an unchanged drug [67]. Thus, the TA dose should be adjusted according to the patient’s renal function, and side effects should be closely monitored in this special population [68].

### 2.3. Nasal Telangiectasia, Ablative Therapies, and Surgical Procedures

Ablative therapies include laser photocoagulation treatment (CO_2_, argon, neodymium-doped yttrium aluminum garnet, flashlamp-pulsed dye, and potassium titanyl phosphate), radiofrequency ablation, electrosurgery, and sclerotherapy [69]. Laser therapy is preferred to cauterization due to reduced damage to the nasal mucosa. In a study including 221 international HHT patients, laser therapy was beneficial [70], and it is fully established as the optimal ENT treatment option with decades of use supporting its safety and efficacy [4]. Sclerotherapy, using a polidocanol 1% injection under the mucosa or perichondrium to obstruct blood flow [71], can also be efficient [72], although experts recognize a risk of blindness due to arterial anastomoses [4]. The International Guidelines recommended these procedures as the third-line option for patients who continue to experience significant nosebleeds after moisturization and systemic use of TA. The choice of a specific procedure depends on local expertise, with laser therapy preferred, as highlighted in a recent publication by the European Reference Network (VASCERN) [4]. Some studies have also described the beneficial effects of sclerotherapy using sodium tetradecyl sulfate and polidocanol [69,73]. The rare cases of blindness emphasize the importance of patient counseling for the choice of management [4]. To detail these studies, a crossover prospective randomized trial involving 17 HHT patients with recurrent epistaxis suggested that sodium tetradecyl sulfate sclerotherapy results in a greater reduction in ESS (−0.95, one-sided *p* = 0.027) than standard care (moisturization, cautery) [51]. A retrospective study evaluating 67 patients with HHT suggested that sodium tetradecyl sulfate sclerotherapy is similar in controlling epistaxis as laser cautery with fewer procedures required [74]. Radiofrequency at low temperatures has been considered as another alternative to laser therapy. It was effective in a study of 57 patients with HHT who underwent 150 treatments over the course of 12 years; the average duration of effectiveness was 25 months (range 1 to 86 months) [75]. Thus, due to positive results, ablative therapies are recommended as part of epistaxis management. However, these invasive interventions usually have to be repeated, as their effectiveness is temporal, and all of these techniques carry the risk of nasal septum perforation. Nasal arterial embolization can be effective, but only in the short term, carrying additional risks so it is rarely used [4,70].

Even with the use of systemic antiangiogenic agents to treat recurrent epistaxis, refractory patients can benefit from more invasive surgeries such as septodermoplasty (skin grafting), or unilateral or bilateral nostril closure (Young’s procedure) that lead to epistaxis cessation in up to 83% of patients, with a 4.68 g/dL average increase in the hemoglobin level and a decrease in patient’s needs for transfusion in two large studies [76,77]. The closing of nasal nostrils prevents airflow from damaging the fragile telangiectases in the nose, but there are significant additional consequences such as needing to breathe through the mouth, loss of taste and/or smell, and needing to swallow blood if nosebleeds persist. Thus, despite efficacy, many patients decline or reverse this effective procedure [76,77]. Weighing the Benefits/Risk Ratio, nasal closure and septodermatoplasty are considered for the management of transfusion-dependent and/or QoL-impacting epistaxis that failed to respond to moisturizing, TA, and/or ablative therapies [2].

### 2.4. Bevacizumab

Bevacizumab is a humanized monoclonal antibody that binds the circulating vascular endothelial growth factor-A (VEGF-A), inhibiting it from activating its proangiogenic receptors [78,79]. Originally marketed as Avastin^®^, with biosimilars now available, it was first approved in combination with chemotherapy as a first-line treatment for colorectal cancer to target its neo-vasculature [80], and later indications have included other oncological conditions [80]. The effectiveness of bevacizumab to target the cancer-associated angiogenesis, via VEGF, varies depending on the specific cancer type, stage, and patient characteristics. Interestingly, more than 20 years ago, with HHT described as a model of blood vessel growth and enlargement [81], increased levels of the pro-angiogenic VEGF were hypothesized to be implicated in the physiopathology of HHT [82]. A few years later, Sabbà et al. [83] and Riedel et al. [84] showed that HHT patients have a significantly higher VEGF plasma concentration than healthy controls. Riedel et al. also performed immunostaining of VEGF in nasal tissue and found an increased VEGF expression in HHT patients mostly localized within regions of angiodysplasia [84]. In 2006, a case report describing a 73-year-old HHT patient suffering from cancer provided the first evidence for the use of bevacizumab in HHT management [85]. The patient received bevacizumab (5 mg/kg every 2 weeks) as part of his anticancer therapy before showing a decrease in the need for transfusion and an increase in his hemoglobin concentration [85]. Since then, evidence in favor of bevacizumab use in HHT started to gather from a series of small reports and trials with consistent yet weak data [86,87]. Based on early results and case reports, and due to the lack of available treatments for severe hepatic forms of HHT, a single-center, phase 2 trial was organized to test the efficacy of bevacizumab on 25 HHT patients with severe hepatic AVMs associated with increased cardiac output [88]. In this preliminary study, bevacizumab significantly decreased cardiac output and mean epistaxis duration per month and improved patients’ QoL [88], with hemoglobin levels remaining stable [88]. The reported side effects in this trial were grade 3 systemic hypertension, headache, nausea and vomiting, asthenia, abdominal and muscular pain, diarrhea, and rash [88]. With the perspective of treating refractory HHT-related epistaxis and avoiding bevacizumab-induced side effects, a prospective, open-label, non-comparative study enrolled six patients to receive a low dose of 0.125 mg/kg of bevacizumab once per month for six months [89]. While the ESS seemed to improve over the course of treatment, there was no change in hemoglobin levels or hematocrit levels, and side effects such as headache or change in taste sensation were reported [89]. Nevertheless, more recent data suggest that bevacizumab at 5 mg/kg may improve hemoglobin levels of HHT patients with symptomatic hepatic involvement [79,90,91]. The 2021 InHIBIT-Bleed study, an international multicenter retrospective study, compared the effect of bevacizumab with prior treatments in 238 patients [92]. In this study, bevacizumab increased the mean hemoglobin level by 3.2 g/dL and decreased the ESS by 3.4 points [92]. The only double-blind, multicenter, randomized trial available evaluating the safety and efficacy of bevacizumab on severe bleeding in HHT patients was published in 2023 [38]. It was a small trial in which 24 HHT patients with a high transfusion requirement received bevacizumab 5 mg/kg (every 14 days for six infusions) or placebo. Because the trial was underpowered, the primary outcome—the comparison of success rates defined as a ≥50% reduction in red blood cell (RBC) transfusions—did not reach statistical significance (63.6% vs. 33.3%). Nevertheless, the findings are considered clinically relevant, as the number of RBC transfusions decreased by 44% in the bevacizumab group compared with only 4% in the placebo group. Moreover, a statistically significant increase in hemoglobin levels was observed in the bevacizumab group relative to the placebo group [38]. When the European Reference Network on Rare Multisystemic Vascular Diseases (VASCERN) evaluated the adverse effects of bevacizumab in a 2019 study including 69 HHT patients [93], the most common adverse effects reported were joint pain (10%), headache (4%), and proteinuria (3%). They reported two patients who experienced major bleeding: one was grade 3 gastrointestinal bleeding, the other was fatal hemoptysis from an unmonitored PAVM. This was considered possibly related to bevacizumab, as the spontaneous rupture of PAVMs was unlikely in this patient [93]. Other potential toxicities of bevacizumab identified in the general population include cardiovascular effects (hypertension, thromboembolism, and left ventricular dysfunction), non-cardiovascular effects (delayed wound healing, gastrointestinal perforation, fatigue, and dysphonia), and fatal adverse events.

Most clinical use is of systemic bevacizumab administered intravenously; however, a small study investigated the use of intranasal bevacizumab by submucosal injection or by topical spray [94]. A case series suggested promising results, although the North American Study of Epistaxis in HHT showed no significant differences between topical bevacizumab and placebo [43]. Two clinical trials, conducted to test the safety and efficacy of bevacizumab nasal spray, showed good tolerance but no efficacy in treating HHT epistaxis [40,41]. Furthermore, a double-blind, randomized, placebo-controlled trial conducted on 15 HHT patients with a minimum of two epistaxis episodes per week at the Medical University of Vienna analyzed the efficacy of one intramural injection of 100 mg of bevacizumab and showed no statistical differences between the two groups [42]. Likewise, bevacizumab submucosal injection did not show any statistical difference in ESS with saline upon a single dose administration during electrocautery [95].

In light of the currently available data, the use of systemic bevacizumab for the management of HHT-related epistaxis is recommended for patients that have failed to respond to moisturizing topicals, TA, and/or ablative therapies [2]. A recent study on the cost effectiveness of bevacizumab therapy reports that bevacizumab therapy saved patients an average of 133 h spent receiving HHT-specific care per year of life, thus concluding that bevacizumab should be considered for more favorable formulary placement in the care of patients with moderate-to-severe HHT [96]. Bevacizumab was designed as an orphan drug (EU/3/14/1390) for HHT in 2022 by the European Medicine Agency (EMA) (Table 2).

## 3. Treatment Options Under Investigation

Given the limited availability of safe and efficacious treatment modalities within the evidence-based recommended approaches, scientists and physicians continue to explore novel alternatives. Some of these arise from serendipitous observations, where patients with HHT experienced epistaxis improvement after receiving a medication intended for an unrelated condition. Others stem from the targeted repurposing of drugs based on their known or newly discovered antiangiogenic effect. While many show optimistic outcomes, none have yet to obtain approval, primarily due to the lack of randomized control trial evidence or side-effect profiles that are not considered to outweigh potential clinical benefit. Below we discuss the advancement and potential role of each treatment in the management of HHT-related epistaxis, highlighting novel scientific insights that rationalize their use.

### 3.1. Immunomodulatory Imide Drugs (IMiDs): Thalidomide and Derivatives

Thalidomide, mostly known for its antiangiogenic properties, is the pioneering IMiD drug approved for managing conditions characterized by excessive angiogenesis, such as tumor vasculature [97] and AVMs in HHT. In a 2010 publication, Lebrin et al. shed light on one of the mechanisms by which thalidomide acts to inhibit HHT-related epistaxis [98]. In their study, the oral daily administration of 100 mg of thalidomide significantly decreased the frequency and duration of epistaxis in HHT patients within 1 month of treatment. Importantly, thalidomide enhanced hemoglobin levels and decreased the need for transfusion. Lebrin et al. showed that thalidomide is capable of increasing endothelial platelet-derived growth factor-B (PDGF-B) paracrine signaling, which has a role in recruiting vascular mural cells, in addition to a direct effect on mural cells’ behavior, both leading to the rescue of the abnormal vascular wall characteristic of HHT [98].

Thalidomide is also a potent anti-inflammatory drug capable of decreasing tumor necrosis factor-α (TNFα) protein levels by binding and destabilizing its mRNA [99,100]. Interestingly, inflammation is proposed as a key driver in AVMs development [101], and cytokines such as TNFα were proposed to induce a transient and local null-endoglin phenotype during inflammation [102]. Thus, three possible mechanisms of action for thalidomide in HHT are considered: (i) antiangiogenic effect; (ii) pericyte recruitment and vessel wall stabilization; and (iii) anti-inflammatory effect. However, despite these promising mechanisms of action, the therapeutic potential of thalidomide in HHT is blunted by its side-effect profiles [103,104]. Notably, thalidomide-related adverse events were shown in a study of VASCERN-HHT to be more common in *ENG* (17/17) than in *ACVRL1* (14/34) patients [93]. The follow-up of a cohort including all HHT patients treated with thalidomide in the Netherlands between 2009 and 2014 showed that approximately one-third of the patients discontinued the therapy as a result of serious adverse events that outweighed the benefits on HHT-related bleedings, and none had remained on the drug despite efficacy [103]. In this study, the most commonly reported adverse effect was neuropathy, followed by a general malaise in half of the patients. Severe skin reaction, as well as pain and edema, were also strong enough to stop the thalidomide treatment. Additionally, dizziness and drowsiness, tremor, mood changes, immunodepression, dyspnea, and gastrointestinal symptoms were reported in severe forms [103]. Even when administered at an initial low dose of 25 mg twice daily, followed by a gradual increase of 25 mg/day every week, thalidomide still had to be discontinued in 2/7 of HHT patients due to severe adverse events, with 4/7 reporting mild to moderate side effects [104]. Recent systematic reviews confirm that thalidomide showed a significant reduction in the frequency and duration of HHT nosebleeds, unfortunately, with a high frequency of considerable adverse events [97,105]. Thalidomide was designed as an orphan drug for HHT in 2017 by the EMA (EU/3/17/1845) (Table 2). Notably, to reduce the need for systemic administration, thalidomide nasal powders have been developed with the perspective of an adjunct therapy and are in preclinical development [106].

Lenalidomide and pomalidomide, both derivatives of thalidomide, represent the second and third generations of IMiDs, respectively [107]. Lenalidomide was developed with the aim of having a more potent inhibitor of TNFα production [108]; it also showed more potent antiangiogenic activity in vitro, accounting for its inhibition of cellular migration [109]. A case report suggested that it could alleviate HHT-related AVMs and bleeding with better tolerance compared to its first-generation analog [110]. Likewise, pomalidomide was developed to have enhanced anti-inflammatory potential with a better safety profile. A pilot study suggested that pomalidomide decreases angiogenic markers in HHT patients, including VEGF-A, VEGF-C, Angiopoietin 1, and Heparin Binding EGF-Like Growth Factor (HB-EGF) [111]. Recently, the results of PATH-HHT, a randomized, multicentric, placebo-controlled clinical trial evaluating the safety and efficacy of pomalidomide in treating HHT, were published. It included 144 HHT patients that were randomized at a 2:1 ratio to receive either 4 mg of daily pomalidomide or placebo for 6 months. The drug was reported to significantly decrease ESS values and the HHT-specific QoL score and increase hemoglobin with effects comparable to bevacizumab. Unlike its first-generation analog, pomalidomide did not induce peripheral neuropathy [112]. However, neutropenia was induced in almost half of the patients that received pomalidomide. Also, gastrointestinal perturbations, rash, and venous thrombosis were reported [112]. More recently, some concerns regarding the reported pomalidomide effects in HHT were expressed by different investigators [113]. Among others, the reported efficacy of pomalidomide was considered to be modest and independent of genotype (*ENG* or *ACVRL1*) [113]. In addition, it was suggested that the high cost of pomalidomide and its limited efficacy do not seem to justify displacing thalidomide in future studies of treatments for HHT [113].

### 3.2. Kinase Inhibitors

#### 3.2.1. Pazopanib and Nintedanib

**Figure 3:**
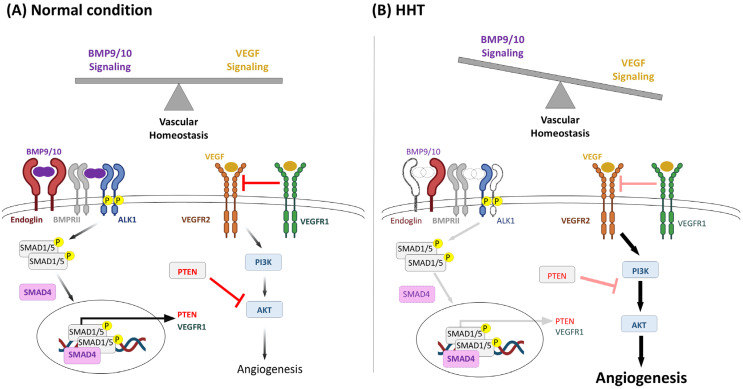
BMP9/10 and VEGF signaling crosstalk in HHT. BMP9/10/ALK1 signaling regulates the expression of endothelial genes, including the decoy VEGF receptor VEGFR1 and the phosphatase and tensin homolog (PTEN). VEGF activates its VEGFR2 Tyrosine Kinase Receptor, which signaling leads to downstream PI3K and AKT activation, favoring angiogenesis. In non-pathological conditions (**A**), BMP9 balances VEGF signaling by favoring vascular quiescence genes and upregulating inhibitors of VEGF signaling including PTEN and VEGFR1. Heterozygous loss of function of endoglin, ALK1, SMAD4, or BMP9 in HHT (**B**) leads to overactivation of VEGF signaling and excessive angiogenesis. Adapted from Queisser et al. [114].

Phosphatidyl Inositol 3-Kinase (PI3K)/AKT/mTOR is a major angiogenesis and vascular growth pathway, where pathway-activating mutations cause multiple non-HHT vascular anomaly syndromes (Figure 3) [114]. Overlaps with HHT were suspected because BMP9 signaling negatively regulates PI3K/AKT signaling through PTEN activation [115]. Additionally, *Alk1* deficiency in mice leads to vascular hyperplasia concomitantly with an upregulation of PI3K signaling [115,116]. The vascular phenotype of the *Alk1*-deficient mice could be prevented and/or rescued by the administration of Wortmannin, a PI3K inhibitor [116], or by heterozygous crossing with constitutive kinase-dead PI3K mice [115]. Furthermore, immunochemistry analysis of cutaneous telangiectasia biopsies shows increased PI3K activation in HHT1 [117] and HHT2 [115] patients’ vessels. Thus, HHT-induced alterations in the BMP9/ALK1/ENG/SMAD4 signaling pathway seem to crosstalk and upregulate the VEGF/Tyrosine Kinase Receptors (TKR)/PI3K/AKT proangiogenic signaling. Therefore, TKR, PI3K, and AKT are considered to be potential targets in the management of HHT (Figure 3). Pazopanib belongs to a family of Tyrosine Kinase Inhibitors (TKIs) which target the three VEGF receptors and the PDGF α and β receptors, in addition to c-kit. The TKI drugs were developed for targeted antiangiogenic cancer therapies [118]. As the inhibition of VEGF with bevacizumab was not suitable nor efficient for all HHT patients, in 2017, Kim et al. explored the potential of inhibiting the angiogenic pathway by targeting the VEGF receptor with oral TKIs and showed that sorafenib, a pazopanib analog, improved hemoglobin levels in an Alk1-depleted mouse model [119]. A 2018 clinical trial, which was being conducted to test the safety and efficacy of pazopanib for HHT hemorrhagic and anemia, had to cease due to the sale of the pazopanib portfolio to another pharmaceutical company. An already enrolled 61-year-old patient, whose epistaxis failed to respond to bevacizumab, purchased pazopanib privately before a Young’s procedure [120]. Remarkably, his ESS and hemoglobin levels improved, and his need for red blood cells transfusion decreased within one month of taking 50 mg of pazopanib per day. After a well-tolerated increase to 100 mg daily, his hematological parameters continued to raise as he continued the therapy for one year [120], and a Young’s procedure was avoided. The use of pazopanib for HHT-related epistaxis was also assessed in a prospective multicenter, open-label, dose-escalating trial [49]. Together with a retrospective review of 13 transfusion-dependent HHT patients [121], it has been concluded that pazopanib decreases epistaxis severity and duration and increases hemoglobin levels in most patients [49,121]. The most commonly reported side effects in the retrospective study were hypertension, lymphocytopenia, and fatigue, with 92% of patients experiencing at least one adverse event [121]. In the proof-of-concept study, one patient interrupted treatment due to hepatotoxicity detected by elevated liver function tests [49]. Thus, the available data regarding the use of pazopanib in the management of HHT epistaxis warrants further study. Currently, a randomized, placebo versus control, double-blind phase II/III trial (NCT03850964) is evaluating the effects of a low dose (150 mg) of pazopanib taken daily for 24 weeks on epistaxis and anemia in HHT. The completion of this trial is estimated to be around 2026.

Nintedanib, another TKI, was also recognized as effective in HHT following incidental administration at 150 mg PO twice daily to a 70-year-old male HHT patient to treat his newly diagnosed idiopathic pulmonary fibrosis (IPF) [122]. Besides stabilizing the patient’s pulmonary disease, nintedanib dramatically decreased his ESS from 5.53 to 0.51. Prior to TKI initiation, he reported multiple daily epistaxis lasting from 6 to 15 min each. After 3 weeks of therapy, he was subject to one epistaxis per month, which lasted without worsening during 12 months of follow-up [122]. Consequently, the EPICURE study (NCT03954782) was performed in France with the aim of evaluating the efficacy of 150 mg of oral nintedanib twice daily. In the 2024 HHT international meeting, the results of the phase II, double-blind, placebo-controlled trial were presented. In 60 patients that were randomized, 30 to placebo and 30 to nintedanib, for 12 weeks, results were promising in the reduction in the duration and frequency of epistaxis, as well as in hemoglobin levels, but did not reach statistical significance (a reduction of more than 50% in the duration of epistaxis was sought). The differences between nintedanib and placebo in the QoL measured by the SF36 scale also did not reach significance. Diarrhea was the most common adverse effect, which lead to the discontinuation of treatment in some patients [48].

#### 3.2.2. VAD044

Vad044 is a new allosteric inhibitor of AKT developed for the treatment of HHT. An ongoing trial is being been sponsored by the company Vaderis^®^ after a proof-of-concept study with two doses of VAD044: 30 and 40 mg once a day for 3 months [123]. Epistaxis, hemoglobin, and QoL were assessed. A total of 75 patients from various European countries were randomized, with 25 patients treated with placebo, 18 with 30 mg doses, and 26 patients with 40 mg doses. There was no significant difference in ESS monitored using a digital application between 30 and 40 mg, although a significant difference of 1.6 points was observed in ESS compared to placebo. A regression of cutaneous telangiectases after 12 months was observed in patients that received 40 mg of VAD044, and the increase in QoL was statistically significant [123]. Few adverse effects were reported, including hyperglycemia in two cases, and the most common was cutaneous urticaria. Currently, evaluation of VAD044 efficacy to treat HHT patients in a phase 1b clinical trial is ongoing (NCT05406362). Because the preliminary data are encouraging, on 18 November 2024, Vaderis^®^ received a Food and Drug Administration (FDA) Fast Track Designation for VAD044 for the treatment of HHT, underscoring its potential [124].

#### 3.2.3. Trametinib

Trametinib is a mitogen-activated extracellular signal-regulated kinase 1 (MEK1) and MEK2 inhibitor that is currently used in the clinic to treat different types of cancer. An HHT1 patient with an unrelated gynecologic low-grade cancer received trametinib (1 mg/day), and the tumor responded well for more than 2 years. Interestingly, at the same time, the patient’s nosebleeds were reduced in intensity and frequency. Also, regular red cell transfusions to treat anemia were reduced from every 3 weeks at one point to >12 weeks [18]. The patient had initially received 2 mg daily which was rapidly decreased to 1 mg due the development of hand edema. At this dose, no serious adverse effect was reported [18]. The trametinib targets, MEK1 and MEK2, are dual-specificity serine/threonine and tyrosine protein kinases that, once activated by RAF kinases, phosphorylate extracellular signal-regulated kinase (ERK) that regulates fundamental cellular processes governed by mitogen-activated protein kinase (MAPK) signaling pathways [125,126]. Mechanistic support for the activation of the MAPK pathway in HHT was provided from RNA-sequencing data from blood outgrowth endothelial cells (BOECs) isolated from the control and HHT donors with heterozygous mutations in *ACVRL1*, *ENG*, or *SMAD4* [18]. Additionally, in vitro studies show that endoglin expression inhibits the MAPK pathway, including the phosphorylation of ERK, a target of MEK, while endoglin silencing stimulates the levels of phospho-ERK in epidermal cells [127], and endoglin, via its interaction with β-arrestin2, inhibits the TGF-β-mediated ERK activation [128]. Together, these results support the potential role for MEK1 inhibition to reduce morbidity from HHT-associated hemorrhage and anemia.

#### 3.2.4. Beta Blockers

Beta blockers are a class of molecules that are widely used in cardiovascular diseases, particularly for lowering both the pulse and the stroke volume, by antagonizing beta-adrenergic receptors. In 2008, propranolol, a nonselective beta blocker, was proposed to also display an antiangiogenic effect as it improved infantile hemangiomas (IH) in pediatric patients [129]. IH is a proliferative vascular disorder in which endothelial cells, pericytes, dendritic cells, and mast cells form benign tumors [129]. In addition to the expected vasoconstriction, propranolol inhibited VEGF signaling, which is a key driver of hemangioma growth [130]. Noting that propranolol is a cheap and widely available drug that exhibits a good safety profile in comparison to other antiangiogenic agents, it was proposed to be repurposed for further vascular disorders in which VEGF is implicated including HHT [31]. Albiñana et al. demonstrated that propranolol is able to decrease cellular migration and tubulogenesis of endothelial HHT cells, suggesting it as an alternative antiangiogenic treatment of the disease [131], although propranolol also showed fibrinolytic activity that can worsen bleeding, especially at telangiectasia sites [131]. Propranolol was tested by topical administration in HHT patients [132,133]: in 2020, a placebo-controlled randomized trial assessed the safety and efficacy of 1.5% propranolol gel applied twice daily in each nostril of HHT patients on the severity of epistaxis. The intervention showed a significant improvement of the ESS and hemoglobin levels in the propranolol group with no changes in nasal endoscopy findings. The reported side effects were a burning sensation in the nasal mucosa and rhinorrhea [133]. Timolol, another nonselective beta blocker, was tested in the form of a nasal spray [56] and intranasal thermosensitive gel [57] as novel HHT-related epistaxis treatments. However, both formulations failed to demonstrate significant improvement over the placebo.

#### 3.2.5. Itraconazole

Itraconazole, a widely utilized and affordable drug, illustrates another case of repurposing upon discovery of a novel property entirely distinct from the drugs’ established mechanism of action. This TKI is an antifungal agent which has been used in clinical practice for more than three decades [134]. During a screen for human endothelial cell proliferation inhibitors, itraconazole unexpectedly emerged as a promising antiangiogenic agent [135]. Upon in vivo confirmation of the antiangiogenic effect, it was suggested that the drug’s novel property stemmed from its ability to inhibit VEGFR glycosylation, significantly affecting its autophosphorylation following VEGF stimulation, and consequently leading to the suppression of VEGF signaling [134]. Translationally, a pilot study was designed on 21 HHT patients to investigate the potential benefit of an oral administration of itraconazole 200 mg in alleviating nosebleeds [47]. As reported, the intervention was able to decrease the monthly epistaxis frequency and duration significantly. However, the hemoglobin levels were unchanged, and the improvement in QoL was modest [47]. There was a wide spectrum of reported adverse events including gastrointestinal symptoms, hepatotoxicity, skin rash, neurological symptoms, and cardiac effects. Approximately 20% of patients on itraconazole ended their therapy due to perceived side effects.

#### 3.2.6. Etamsylate

Etamsylate/Dobesilate, a widely used hemostatic agent [136], was discovered to have antiangiogenic properties mediated by the inhibition of fibroblast growth factor (FGF) signaling. In 2019, Albiñana et al. showed that etamsylate decreases in vitro migration and tubulogenesis of endothelial cells, derived from HHT2 patients. Mechanistically, the treatment of cells with this compound decreased the activation of AKT and ERK signaling, both implicated in the physiopathology of HHT. Notably, etamsylate did not show toxicity at the used doses [137]. A pilot clinical trial (EudraCT: 2016-003982-24) evaluated the use of a twice daily topical application of etamsylate spray on epistaxis for 12 HHT patients for 1 month. The study had many limitations, including the use by some patients of propranolol or raloxifene, which may decrease epistaxis, or anabolic steroids or acenocoumarol, which may increase bleeding. Furthermore, the contribution of the placebo effect was unknown [137]. Yet, as the mean ESS decreased, and considering the high safety profile of this intervention, topical etamsylate obtained the orphan drug designation for HHT epistaxis (Table 2).

### 3.3. Treatments Targeting Endoglin and ALK1 Haploinsufficiency

#### 3.3.1. Hormonal Treatments

The use of estrogens in HHT was proposed in the 1950s based on interesting observations in which both high and low estrogen were linked to nosebleeds as women described their epistaxis changes through the menstrual cycle and menopause [138]. For example, Koch et al. described a female HHT patient whose epistaxis happened during the five days preceding her menstrual period and stopped on the first day of her new cycle [138]. At the age of 42, this woman was subject to an artificial castration by radiation of the ovaries that immediately led to an increase in the incidence and severity of her epistaxis and telangiectasia. These symptoms were alleviated by the administration of ethinyl estradiol (EE) 0.5 mg twice daily and came back each time the patient discontinued the medicine due to its heavy side effects [138].

At this time, EE was the first estrogen to be administered to HHT patients before the identification of causal genes [138]. In an uncontrolled study of five HHT patients whose symptoms seemed to improve, side effects including ankle edema, weight gain, and swelling and soreness of breasts forced the interruption of the hormonal therapy. Following the first study, some trials were conducted to better evaluate the efficacy of hormonal treatments [139]. A randomized double-blind trial including 31 HHT patients showed a slight but not significant reduction in epistaxis frequency between the placebo and patients who received 4 mg of estrogen valerate [140]. Further studies showed that high doses of estrogen were beneficial but associated with considerable side effects [141]. For women, besides nausea, breast tenderness, and weight gain, the high-dose estrogens were shown to cause thromboembolic disease with an increased risk of death [141] and as a result have been removed from general medical practice where 20–30 µg dosages are the norm. These have not been tested in HHT to date as they would remain unsuitable for male patients who experience loss of libido (in one case, treated by combining methyl testosterone to EE) [138]. Topical estrogens were tested in combination with argon plasma coagulation and seemed to extend the bleeding-free period [142,143].

Mechanistically, the identification of *ENG* and *ACVRL1* as the first HHT genes provided support for the observed efficacy of estrogens. A proposed mechanism involved the hormones’ signaling in which estrogen binds to the estrogen receptor (ER), forming a complex that recognizes estrogen-responsive elements (EREs) in the DNA, increasing the transcription of specific target genes [144]. An estrogen-responsive element was found in the 5′-flanking region of the *ENG* gene, suggesting a transcriptional control of *ENG* by estrogens [145]. Additionally, raloxifene, a Selective Estrogen Receptor Modulator (SERM) capable of activating ER, increased the expression of endoglin and ALK1 at the surface of endothelial cells with an increase in the genes’ promoter activity [146]. Therefore, by increasing the transcription of the functional allele, estrogen agonists are proposed to counteract the haploinsufficiency of *ENG* and *ACVRL1* genes, whose mutations account for over 90% of HHT cases [147].

Many formulations including estrogens, estrogen/progesterone, SERMs (tamoxifen, raloxifene), ethinyl estradiol/norethindrone, and danazol [148] have been tested on HHT patients. Tamoxifen was first used in 2001: Zacharski et al. reported a case of a 78-year-old HHT female patient who was diagnosed with a hormone-dependent breast cancer treated with tamoxifen [149]. Shortly after tamoxifen initiation, her HHT-related gastrointestinal bleeding seemed to cease and her hematological parameters improved [149]. Following shortly after, a similar case was described in which a 57-year-old women treated with tamoxifen for a hormone-dependent breast cancer had her epistaxis go from uncontrolled/severe to almost non-existent upon the start of tamoxifen [150]. Two RCTs comparing the efficacy of 20 mg/day of tamoxifen administered orally over placebo in treating HHT epistaxis showed improvement in the frequency and severity of bleeding, with some regression of telangiectasia and tolerable side effects in males [53,54]. It is unclear why these papers [53,54] were not included in the evidence reviewed by the expert panels for the Second International Guidelines, particularly as this drug has been used in millions of women to prevent breast cancer. At present, tamoxifen remains widely used for troublesome HHT epistaxis that has not responded to laser treatment in the UK and many other countries, where an issue has become the reluctance of female patients to stop after 5 years as recommended (due to risks of uterine lining hyperplasia).

Raloxifene (Evista^®^), currently approved for postmenopausal osteoporosis management, was assessed in a prospective study on 19 HHT patients [151]. According to a questionnaire-based evaluation, raloxifene seemed to decrease epistaxis severity, and, in 2010, the EMA and FDA granted the orphan drug designation for raloxifene (2010 EU/3/10/730) (Table 2), allowing its use in HHT management [152]. There are side-effect profiles in addition to known protection against osteoporosis, with postmenopausal women displaying an increased risk for coronary events and venous thromboembolism [153]. Similarly to raloxifene, bazedoxifene acetate (Pfizer, New York, NY 10001, US), a third generation SERM, significantly decreased the frequency and intensity of epistaxis while improving hemoglobin levels as early as one month after treatment with 20 mg/day [154,155]. Bazedoxifene was able to increase *ENG* and *ACVRL1* gene expression levels, as demonstrated not only by in vitro experiments using endothelial cells but also in vivo when analyzing macrophages derived from patients treated with the drug. Bazedoxifene was designed as an orphan drug for HHT in 2014 by the EMA (EU/3/14/1367) (Table 2).

#### 3.3.2. Tacrolimus and Sirolimus

The choice of testing tacrolimus in HHT emerged from the compelling observation of a woman with HHT who had undergone a liver transplant and was treated with the immunosuppressor agent to prevent the rejection [156]. Two months after transplantation, her HHT symptoms improved as her skin and mucosal telangiectasia disappeared and her anemia normalized. While many other parameters regularized after liver transplantation including cardiac output, at the molecular level, Albiñana et al. demonstrated that tacrolimus is capable of increasing *ENG* and *ACVRL1* mRNA and protein levels in cultured endothelial cells [157]. Hence, tacrolimus may specifically target the genetic cause of HHT. The safety and efficacy of two tacrolimus formulations, a 0.1% nasal ointment [52], and oral tablets [158] were tested. The nasal ointment was evaluated against the placebo in a randomized trial in which HHT patients self-applied into each nostril the medicine twice daily using a cotton swab for 6 weeks. The toxicity was very low, as no adverse event was reported. However, tacrolimus ointment failed to achieve the primary outcome, which was a significant decrease in the mean epistaxis duration after tacrolimus treatment [52]. In the open-label pilot study, tacrolimus was administered in order to obtain a trough level between 2 and 3 µg/L. Interestingly, hemoglobin levels significantly increased at the end of the trial but not in patients suffering from epistaxis alone or gastrointestinal blood loss alone. At least one adverse event was reported in 64% of the patients. There were two serious adverse events, a neck abscess with subsequent *Staphylococcus aureus* bacteremia and acute lymphatic leukemia [158]. Recently, a phase II open-label clinical trial evaluated low-dose tacrolimus for epistaxis in HHT (NCT04646356). The trial was conducted with non-immunosuppressive low-dose oral tacrolimus (between 2 and 5 ng) in a single center, and patients were followed for 3 months before treatment and for 6 months after. The bleeding measure used was the cumulative weekly bleeding (PROCB) recorded by each patient, measured in minutes and recorded in a patient diary. Every 2 weeks the PROCB for each patient was recorded by the investigator. In addition, variables such as ESS, hemoglobin, red blood cell count, and iron infusions required, as well as adverse effects, were recorded. The results were a decrease in PROCB by 15.9 min, a decrease in ESS by 1.28, and improved hemoglobin levels. There were no serious adverse events [159].

Sirolimus, a macrolide analog of tacrolimus, was used as part of an immunosuppressive protocol in a suspected HHT patient. Reported in 2006 by Skaro et al., the use of sirolimus along with aspirin seemed to decrease vascular malformations and mucosal hemorrhage [156]. Compared to tacrolimus, sirolimus also seemed to rescue the defective SMAD1/5/8 observed in HHT alongside the beneficial inhibition of mTOR overactivation in HHT endothelial cells [160]. An open-label, pilot phase 2 study consisting of treating HHT patients with daily 2 mg of sirolimus has been performed with 10 patients to assess the safety and efficacy of the drug in HHT (NCT05269849), although the final results remain to be published.

#### 3.3.3. Nitric Oxide Targeting for HHT1

Endoglin has been shown to stabilize the endothelial NO synthase (eNOS), which is responsible for NO production, leading the group of Michelle Letarte to postulate that endoglin deficiency in HHT1 results in the inefficient eNOS coupling of oxygen and L-arginine that induces an excessive superoxide O_2_^−^ formation [144]. These released reactive oxygen species would exert a dilatory effect on the surrounding smooth muscle cells that leads to the abnormal dilation hallmark of HHT. Interestingly, they showed that the elimination of the oxygen reactive species by treatment of the vessels with an antioxidant was capable of reversing the vasomotor abnormalities in a model of HHT [161]. Thus, based on their results, a pilot study investigated the potential of N-acetylcysteine (NAC), a safe and widely used antioxidant, to treat HHT-related epistaxis [162]. It consisted of orally administrating 600 mg of NAC three times daily for 3 months to 43 HHT patients. In line with this hypothesis, HHT1 patients, but not HHT2 patients, significantly responded to NAC therapy [162].

## 4. Discussion and Future Trends

Epistaxis is the most common and disabling manifestation of HHT. Yet, to date, no drug has earned full FDA or EMA approval for its treatment. Interestingly, all pharmacological interventions, whether recommended or under investigation, consist of repurposed drugs that are marketed for indications other than HHT. Novel therapies [163] are progressing through preclinical development, safeguarded by confidentiality in academic or commercial sectors. Searched Primary Registries of the ICTRP Network and partner registries (including clinicaltrials.gov) as of July 25, 2025, contain a single phase I trial testing a novel drug for HHT (VAD044; registration number NCT05406362; Table 1). Many investigated drugs did demonstrate potential and seven earned the “orphan drugs” designation in Europe and/or USA (detailed in Table 2). Among the six existing drugs that were granted the orphan drug status by EMA, three initially received orphan designation under the sponsorship of academic institutions, which subsequently transferred the designation to pharmaceutical companies. At present, no entity (commercial or academic) has successfully completed the regulatory approval process of either the EMA or FDA to include the indication of HHT on the label of the marketed product, and there are many potential reasons.

While the HHT epistaxis drug panel appears extensive and continually expanding, there are only a limited number of options that are widely supported, and the evidence base is weak. In part, this reflects the lack of funding to conduct trials of drugs where a commercial company does not stand to benefit. Additionally, the field is hampered by considering all HHT patients to be equivalent, neglecting their varying HHT manifestations and comorbidities, main HHT causal genes, as highlighted in 2024 [113,163], and the common presence of genetic modifiers that make some individuals with HHT more prone to bleeding [164]. No studies have stratified HHT patients according to possession of high-impact variants in genes affecting bleeding or coagulation. Modarresi et al. reported that, of the 27 HHT drug trials listed on PubMed, only two trials considered HHT-causal genotypes. One of them showed a marked benefit of NAC in *ENG* patients that was masked by the lack of efficacy in *ACVRL1* patients [162]. Of the 41 trials listed on clinicaltrials.gov, only 1 listed the causal HHT genotype as one of multiple secondary outcome measures [163]. The same group pointed out that the pomalidomide study results may also have been more positive if HHT subtypes had been considered [113]. They also suggested a further subdivision of patients based on whether the pathogenic gene variant generates a premature termination codon (PTC), as in frameshift and nonsense mutations that represent almost half of the HHT genotypes [113]. In turn, this raises the possibility that a subgroup of HHT patients may benefit from a separate approach to suppress the formation of potentially proteotoxic species [165]. This is based on evidence from HHT patient-derived BOECs that show remarkable adaptation to their haplo-insufficient state at rest [11,166,167] but abnormal behavior in non-resting states [166]. Second, there is evidence that the PTC-containing transcripts are not fully degraded by nonsense-mediated decay (NMD) but persist at levels of up to 34%, predicted without producing truncated protein but with circumstantial evidence supporting ribosomal readthroughs to generate aberrant full-length proteins [11]. These authors showed that PTC persistence was greater in stressed BOECs, and although stress inhibits NMD, there was evidence that aberrant proteins could induce cell stress by an activating transcription factor-4 (ATF4) readout [11]. Coupled to their clinical data in three cohorts totaling 708 patients who had been phenotyped before genotypes were available [11,12,164], where PTC patients had more severe bleeding and responded better to antibiotic ointments used to reduce inflammation in the nasal mucosa [11], a number of new therapeutic approaches may be considered for this subgroup of HHT patients.

One problem for the field is that treatments are seeking to achieve two different aims: First, a reduction in nosebleeds, which are debilitating for patients and compromise QoL, and second, improvement in hemoglobin, which could be argued to be the more important biomedical parameter. The commonly employed primary measure for determining drug efficacy and treatment response for epistaxis, the ESS, does not consistently demonstrate an enhancement in patient hematological parameters. Indeed, guideline-recommended TA use to treat HHT epistaxis fails to improve hemoglobin levels despite decreasing ESS [85]. Similarly, propranolol nasal gel achieved the primary outcome in a placebo-controlled randomized trial by significantly reducing the ESS (−2.03, *p* = 0.009) but with no notable augmentation in hemoglobin levels reported [133]. Likewise, oral itraconazole diminished the median ESS from 6 to 3.8 (*p* = 0.006) and the monthly duration of epistaxis from 407 to 278 min (*p* = 0.005) and failed to change hemoglobin levels [47]. Thus, numerous instances have been observed where the ESS exhibited significant improvement without a concurrent change in hemoglobin levels. In each case, this raised questions about the efficacy of the treatment option without considering the individual or genotype stratified trends such that effective treatments for many individuals are discarded. Taking into account this specific hematological parameter, the currently available treatment options narrow down to tamoxifen, which is the safest, and RCT, proven to reduce nosebleeds, then thalidomide [55], oral tacrolimus [158], and pazopanib [121], all of which remain under investigation in light of limited evidence of efficacy and notable side-effect profiles.

Other molecules with relatively safe side-effect profiles were tested but seemed to lack proof of efficacy. Doxycycline, a widely available antibiotic, was reported to suppress the activity of the matrix metalloproteinases (MMPs), which degrade the extracellular matrix and activate angiogenic factors [168]. However, it did not demonstrate effectiveness in treating HHT-related epistaxis in two randomized, placebo-controlled trials [44,45].

While most of the drugs used to treat HHT bleeding have been repurposed from the FDA’s and EMA’s arsenals, active basic and translational research may reveal more specific therapeutic targets for HHT. In the first decade of 2000, two different groups described a TGF-β independent role of endoglin in the modulation of the vascular tone [161,169]. Several lines of experimental evidence have supported the role of integrin-dependent cell adhesion of endoglin in the pathogenesis of HHT, including biological processes involved in bleeding [101,170,171,172]. For instance, vessel fragility and increased permeability leading to hemorrhages of mucocutaneous telangiectases could be explained, at least in part, by the impaired binding of endothelial endoglin to integrins in vascular mural cells [171]. Moreover, a deficient binding of endothelial endoglin to leukocyte integrins may impair leukocyte extravasation and, consequently, proper vascular remodeling, leading to vascular telangiectases and AVMs [170]. Also, endothelial endoglin binds to platelets’ αIIbβ3 integrin, in turn regulating endothelial-dependent hemostasis, a process that appears to be deficient in the bleeding associated with HHT [172,173]. Future studies should investigate the potential translation of these experimental findings to a clinical setting.

This review has focused on the pharmacological treatments currently under clinical investigation for HHT-related epistaxis. While it highlights significant advances in managing this condition, it also emphasizes the urgent need for safer and more effective therapeutic options. It is important to remember that overall HHT patients have a normal life expectancy (though this is reduced for those with anemia), and their need for epistaxis treatments can be for many decades. Thus, recent publications which discuss oncology-targeted molecules, including in preclinical studies [174,175], must be tempered with safety concerns, particularly for long-term use in the HHT population. It is unfortunate that, to date, local nasal therapies using current drugs have not demonstrated significant improvement in reducing bleeding compared to placebo, as developing a novel nasal treatment specifically designed to address epistaxis could bypass systemic side effects. Such a therapy could either prevent vascular malformations through antiangiogenic mechanisms or locally activate the hemostasis process to stop bleeding more effectively.

**Figure 4:**
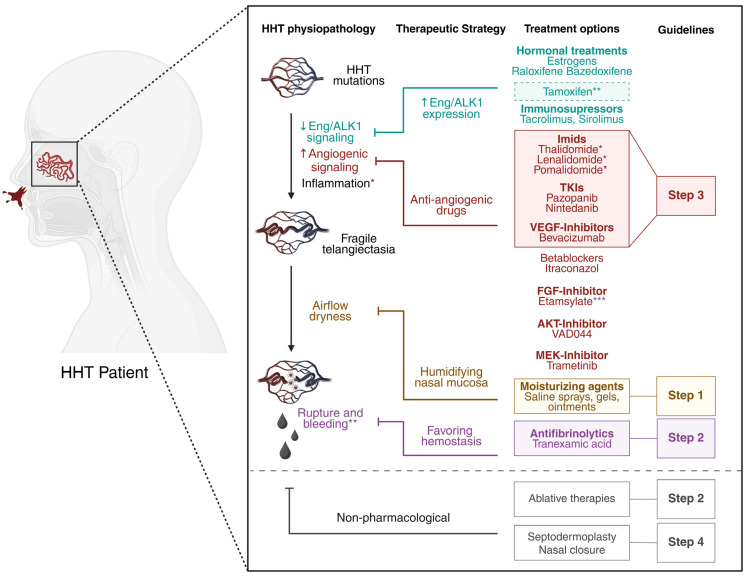
Current treatments for epistaxis in HHT patients. HHT mutations lead to decreased (↓) endoglin and ALK1 signaling and increased (↑) angiogenic signaling, predisposing patients to vessel instability and arteriovenous malformations. Inflammation has been stipulated to be a second hit required for vessel lesions development. The resulting fragile telangiectases at the nasal mucosa, prone to constant airflow, tend to rupture and bleed. Therapeutic strategies involve increasing endoglin and ALK1 expression (in blue), inhibiting overactivated angiogenic pathways (in red), preventing dryness with topical humidifying agents (in orange), and favoring hemostasis (in purple). All molecules shown have been evaluated in clinical trials for the treatment of HHT-related epistaxis. Treatments recommended by international guidelines as first-, second-, and third-line options are highlighted within color-coded squares corresponding to Step 1, Step 2, and Step 3, respectively. Ablative therapies (such as laser therapy and sclerotherapy) and other ENT interventions play an essential role in managing HHT-related epistaxis, as recommended by international guidelines [1,2]. Further details can be found in the primary ENT literature, but a discussion of this specialist field is beyond the scope of the current article. * Imids, in addition of having antiangiogenic properties, are also strong anti-inflammatory drugs. ** Tamoxifen has randomized controlled trial (RCT) evidence of efficacy and is a third-line therapy in the UK and other countries prior to the introduction of antiangiogenic agents, though it was not mentioned in the International Guidelines. *** Etamsylate, known to inhibit FGF signaling, also favors hemostasis. Created with BioRender.com.

In conclusion, this review focuses on current treatments for epistaxis in HHT patients, as well as on new drugs under clinical investigation for managing HHT-related epistaxis (Figure 4). It highlights significant advances in treatment, while also emphasizing the urgent need for safer, HHT-specific, and more effective therapeutic options.

## Figures and Tables

**Table 1:** Interventional clinical trial in HHT *.

ID	Drug/ Intervention	Study Phase	Study Design	Number of Patients	Primary Outcome	Published Results (Peer-Reviewed Journal)
NCT05641142	ANTIPLATELET	Cohort study	Single Group Assignment, Open Label	100	Number of transfusions and/or intravenous iron	N/A
ChiCTR2100043253	BEVACIZUMAB	Phase 4	Single Arm	30	Nose bleeding and SF36 score	N/A
NCT04404881	BEVACIZUMAB	Phase 2	Single Group Assignment, Open Label	33	Change in ESS	N/A
NCT03227263	BEVACIZUMAB	Phase 3	Double-Blind Multicenter Randomized Phase 2 Trial	24	Decrease of at least 50% in the cumulative number of RBC units transfused in a 3-month period	63.6% of patients (7/11) in the bevacizumab group versus 33.3% of patients (4/12) in the placebo group decreased the number of blood transfusions by at least 50% (*p* = 0.22) [38]
NCT02389959	BEVACIZUMAB	Phase 4	Randomized, Parallel, and Quadruple Masking (Participant, Care Provider, Investigator, and Outcomes Assessor)	40	Trial improvement in ESS	N/A
NCT02157987	BEVACIZUMAB	Phase 1/Phase 2	Single Group, Single Masking (Care Provider)	15	Decrease of at least 50% in the number of epistaxis at 1 month of treatment compared to the month prior to inclusion for 60% of patients	Not achieved [39].
NCT02106520	BEVACIZUMAB	Phase 2/Phase 3	Randomized, Parallel Assignment, and Double Blind	75	Mean monthly epistaxis duration for 3 consecutive months immediately after the end of the treatment	No significant difference between treated and placebo group [40]
NCT01507480	BEVACIZUMAB	Phase 1	Single Group, Double Masking (Participant, Investigator)	40	Tolerance of increasing doses of bevacizumab administered as a nasal spray in patients with HHT-related epistaxis	No dose limiting toxicity was observed; no efficacy was observed at any dose in this study [41]
NCT01402531	BEVACIZUMAB	Phase 2	Single Group Assignment, Open Label	10	Epistaxis using the ESS, hematocrit, hemoglobin, and serum ferritin levels	N/A
NCT01397695	BEVACIZUMAB	Phase 2	Single Group Assignment, Open Label	20	Measurement of epistaxis in patients with HHT, as measured by the HHT Foundation ESS, hematocrit, hemoglobin, and serum ferritin levels	N/A
EUCTR2009-018049-19-AT; NCT01314274	BEVACIZUMAB	Phase 2	Double-Blind, Placebo-Controlled Trial	15	Reduction in average visual analog score of epistaxis	No significant change [42]
NCT01408030	BEVACIZUMAB, ESTRIOL, TRANEXAMIC ACID	Phase 2	Randomized, Parallel, and Quadruple Masking (Participant, Care Provider, Investigator, and Outcomes Assessor)	106	Median weekly epistaxis frequency during weeks 5 through 12	Drug therapy did not significantly reduce epistaxis frequency [43]
NCT03397004	DOXYCYCLINE	Phase 2	Randomized, Crossover, and Triple Masking (Participant, Care Provider, and Investigator)	11	Change in weekly epistaxis duration (WED)	No significant difference between the change in WED [44]
NCT04167085	DOXYCYCLINE	Phase 4	Randomized, Crossover, and Double Masking	22	Frequency of epistaxis; duration of epistaxis; and change in severity of epistaxis	No reduction in the three primary outcome measures [45]
EUCTR2016-003982-24-ES	ETHAMSILATE	Phase 2	Open, Single Arm	12	Number of epistaxis per week; bleeding time; amount of bleeding; evolution of anemia; and assessment of QoL	N/A
NCT02638012	FLOSEAL (Hemostatic agent)	Pilot trial	Single Group, Open Label	7	Changes in ESS between baseline and one-month follow-up	No statistically significant difference noted in ESS pre-treatment and one-month follow-up [46]
EUCTR2017-003272-31-NL	ITRACONAZOL	Phase 2	Open, Single Arm	17	Change in ESS	Median ESS decreased during treatment; no change in hemoglobin levels [47]
NCT02963129	MUPIROCIN	Phase 3	Randomized Parallel, Double Blind	40	Nosebleed by Sadick scale	N/A
NCT04976036	NINTEDANIB	Phase 2	Randomized, Parallel Assignment, and Quadruple Masking (Participant, Care Provider, Investigator, and Outcomes Assessor)	48	Change in epistaxis duration	N/A
NCT03954782	NINTEDANIB	Phase 2	Randomized, Parallel, and Triple Masking (Participant, Care Provider, and Investigator)	56	Reduction of at least 50% in mean monthly epistaxis duration	Primary outcome not achieved, but significant decrease in median epistaxis and increase in median hemoglobin levels [48]
NCT04976036	NINTEDANIB	Phase 2	Randomized, Parallel Assignment, and Quadruple Masking	48	Change in epistaxis duration in minutes under nintedanib treatment, as compared to placebo in HHT patients	N/A
EUCTR2018-004179-11-DE	OCTREOTIDE	Phase 3	Controlled, Randomized, Open Label, Parallel Group, and Not Placebo Controlled	38	Decrease of ≥50% in the number of red blood cell transfusions and/or number of IV iron infusions	N/A
EUCTR2018-004179-11-NL	OCTREOTIDE	Phase 3	Randomized, Controlled, and Open	38	Decrease of = 50% in the number of units of intravenous iron and/or blood transfusions given	N/A
NTR7589	OCTREOTIDE	Phase 3	Randomized Controlled Trial, Parallel, and Open	38	Decrease of 50% in the number of units of intravenous iron and/or blood transfusions	N/A
NCT00004327	OCTREOTIDE	Phase 2	Single Group Assignment	8	Unknown	N/A
NCT03850730	PAZOPANIB	Phase 1/Phase 2	Single Group Assignment, Open Label	30	Change in epistaxis duration	N/A
NCT03850964	PAZOPANIB	Phase 2/Phase 3	Randomized, Parallel, and Quadruple Masking (Participant, Care Provider, Investigator, and Outcomes Assessor)	70	Change in epistaxis duration in minutes; hemoglobin response rate increase in hemoglobin	N/A
NCT02204371	PAZOPANIB	Phase 2	Non-Randomized, Single Group, and Open Label	7	Change from baseline in hemoglobin at the indicated time points, and change in epistaxis duration, frequency, and intensity	All seven patients realized a benefit, although of variable individual value [49]
NCT00588146	PEGYLATED INTERFERON ALPHA2B	Phase 2	Randomized, Controlled	10	Change in hemoglobin	N/A
NCT03910244	POMALIDOMIDE	Phase 2	Allocation: Randomized; Intervention Model: Parallel Assignment; Primary Purpose: Treatment; and Masking: Quadruple (Participant, Care Provider, Investigator, and Outcomes Assessor)	144	Change in ESS from baseline through week 24	Pomalidomide significantly decreased epistaxis severity by 0.94 points (*p* = 0.004); neutropenia, constipation, and rash were reported in pomalidomide group [50]
NCT02287558	POMALIDOMIDE	Phase 1	Single Group, Open Label	9	Transfusion requirement measure	N/A
NCT04113187	PROPANOLOL	Phase 3	Randomized Parallel, Quadruple Masking (Participant, Care Provider, Investigator, and Outcomes Assessor)	15	Cumulative duration of epistaxis (in minutes)	N/A
NCT01406639	RANIBIZUMAB	Phase 1	Single Group Assignment, Open Label	10	Epistaxis as measured by the HHT Epistaxis Severity Score (ESS), hematocrit, and hemoglobin and serum ferritin levels	N/A
NCT01408732	SCLEROTHERAPY WITH SODIUM TETRADECYL SULFATE	Phase 1/Phase 2	Randomized, Crossover, and Open Label	17	Change in ESS	Sclerotherapy with STS (vs standard treatment) significantly reduced ESS [51]
NCT05269849	SIROLIMUS	Phase 2	Single Group Assignment, Open Label	10	Electrolytes; hematology; renal function; liver function; ferritin level; blood glucose level; and lipid assessment	N/A
NCT00004654	SOY PROTEIN	Phase 3	Randomized, Crossover	60	Unknown	N/A
NCT04646356	TACROLIMUS	Phase 2	Single Group Assignment, Open Label	30	Reduction in bleeding minutes per week	N/A
EUCTR2019-003585-40-NL	TACROLIMUS	Phase 2	Open, Single Arm	20	Change in hemoglobin level	N/A
NCT03152019	TACROLIMUS	Phase 2	Randomized, Parallel, and Triple Masking (Participant, Care Provider, and Investigator)	49	Percentage of patients experiencing an improvement in their nosebleeds	No significant difference in main objective comparing epistaxis before and after treatment (*p* = 0.77) [52]
NCT00375622	TAMOXIFEN	Phase 2	Randomized, Single Group Assignment, and Double Blind	21	Frequency of epistaxis; duration of epistaxis; and hemoglobin level	Increase in hemoglobin level in tamoxifen-treated patients [53]
NCT00375622	TAMOXIFEN	Phase 2	Single Group Assignment	38	Self-assessment questionnaire of rhinologic QoL and epistaxis grading scale	Bleeding score and the QoL score improved; hemoglobin concentration also improved [54]
JPRN-jRCT2071230066	THALIDOMIDE	Phase 3	Randomized Controlled Trial, Double Blind, Placebo Control, Parallel Assignment, and Treatment Purpose	44	Change in ESS compared to baseline	N/A
JPRN-jRCT2051200141	THALIDOMIDE	Phase 2	Single Arm Study, Uncontrolled Control, and Single Assignment	8	Improvement rate of ESS	N/A
NCT01485224	THALIDOMIDE	Phase 2	Single Group Assignment, Open Label	31	Percentage of patients showing a decrease in the frequency, intensity, and duration of epistaxis and in the blood transfusion requirement	All 31 (100%, 89–100) patients responded to therapy with a significant decrease in all epistaxis parameters (*p* < 0·0001 for frequency, intensity, and duration) [55]
NCT00389935	THALIDOMIDE	Phase 2	Single Group Assignment, Open Label	14	Blood transfusion requirements	N/A
NCT02484716	TIMOLOL	Phase 2	Double-Blind, Placebo-Controlled Study	58	Comparison of mean monthly epistaxis duration 3 months before the treatment and 3 months after the end of the treatment	No significant difference between timolol and placebo group [56]
NCT01752049	TIMOLOL	Proof of concept	Randomized, Single Group, and Quadruple Masking (Participant, Care Provider, Investigator, and Outcomes Assessor)	5	Mean reduction in lesion area (compared with baseline measurement) of treated telangiectasia	N/A
NCT04139018	TIMOLOL GEL	Phase 2	Randomized, Parallel, and Quadruple Masking (Participant, Care Provider, Investigator, and Outcomes Assessor)	23	Change in ESS	No definitive conclusions on the superiority of timolol can be drawn [57]
DRKS00020994	TIMOLOL SPRAY	Phase 2	Randomized Controlled Study, Placebo Controlled, and Crossover	20	Change in ESS	N/A
NCT01031992	TRANEXAMIC ACID	Phase 3	Randomized, Crossover, and Double Blind	23	Change in hemoglobin level within the phases	No significant change [58]
NCT00355108	TRANEXAMIC ACID	Phase 3	Randomized, Crossover, and Double Masking	118	Average monthly duration of epistaxis	Significant decrease in the duration of epistaxis in HHT patients taking TA [59]
NCT05406362	VAD044	Phase 1	Randomized, Quadruple Masking (Participant, Care Provider, Investigator, and Outcomes Assessor)	80	Safety and tolerability	N/A

* The investigation spanned 52 interventional clinical trials, of which 22 have their results published in peer-reviewed journals (until May 2025). ESS, Epistaxis Severity Score; ID, Clinical Trial Registry Identifier; N/A, not available (currently unpublished); QoL, Quality of Life.

**Table 2:** “Orphan drugs” in Europe and US for treating HHT-related epistaxis.

EU ID	FDA ID	Product	Indication	Sponsor Europe	Sponsor USA	Designation Date	Designation Date FDA
EU/3/23/2766	N/A	6-(4-(1-amino-3-hydroxycyclobutyl)phenyl)-1-ethyl-7-phenyl-1H-pyrido [2,3-b][1,4]oxazin-2(3H)-one, L-tartrate salt	Treatment of hereditary hemorrhagic telangiectasia	FGK Representative Service GmbH	Vaderis	20 March 2023	7 December 2022
EU/3/18/2087	N/A	Etamsylate	Treatment of hereditary hemorrhagic telangiectasia	Dobecure S.L.		19 November 2018	
EU/3/17/1845	N/A	Thalidomide	Treatment of hereditary hemorrhagic telangiectasia	PlumeStars s.r.l.	PlumeStars s.r.l.	27 February 2017	19 July 2017
EU/3/14/1390	N/A	Bevacizumab	Treatment of hereditary hemorrhagic telangiectasia	Laboratoires Delbert	Terence M. Davidson, MDUCSD Medical Center and Laboratoires Delbert SAS	16 December 2014	20 December 2022 and 10 December 2022
EU/3/14/1367	N/A	Bazedoxifene acetate	Treatment of hereditary hemorrhagic telangiectasia	Consejo Superior de Investigaciones Científicas (CSIC)		19 November 2014	
EU/3/10/730	N/A	Raloxifene hydrochloride	Treatment of hereditary hemorrhagic telangiectasia	Consejo Superior de Investigaciones Científicas (CSIC)	Consejo Superior de Investigaciones Científicas (CSIC)	10 June 2010	20 August 2010
	N/A	Pazopanib			HHT Foundation International (Cure HHT)		9 October 2019

EU ID (European Union Orphan Drug Identifier); FDA ID (Food and Drug Administration Identifier); N/A: Not applicable (no public formal ID given to FDA Orphan Drugs).

## Glossary

ALK1	Activin Receptor-Like Kinase 1
ATF4	Activating Transcription Factor-4
AVMs	Arteriovenous Malformations
BMP	Bone Morphogenetic Protein
BOECs	Blood Outgrowth Endothelial Cells
EE	Ethinyl Estradiol
EMA	European Medicine Agency
eNOS	Endothelial NO Synthase
ENT ER	Ear Nose Throat Estrogen Receptor
EREs	Estrogen-Responsive Elements
ERK	Extracellular Signal-Regulated Kinase
ESS	Epistaxis Severity Score
FDA	Food and Drug Administration
FGF	Fibroblast Growth Factor
HB-EGF	Heparin Binding EGF Like Growth Factor
HHT	Hereditary Hemorrhagic Telangiectasia
ICTRP	WHO International Clinical Trials Registry Platform
IH	Infantile Hemangiomas
IPF	Idiopathic Pulmonary Fibrosis
IMiDs	Immunomodulatory Imide Drugs
JP-HHT	HHT-Juvenile Polyposis
MAPK	Mitogen Activated Protein Kinase
MEK	Mitogen-Activated Extracellular Signal-Regulated Kinase 1
MMPs	Metalloproteinases
NAC	N-acetylcysteine
NMD	Nonsense Mediated Decay
PDGF-B	Platelet Derived Growth Factor-B
PI3K	Phosphatidyl Inositol 3-Kinase
PTC	Premature Termination Codon
QoL RCT	Quality of Life Randomized Controlled Trial
SERM	Selective Estrogen Receptor Modulator
TA	Tranexamic Acid
TGF-β	Transforming Growth Factor β
TKIs	Tyrosine Kinase Inhibitors
TKR	Tyrosine Kinase Receptors
TNFα	Tumor Necrosis Factor-α
VASCERN	European Reference Network on Rare Multisystemic Vascular Diseases
VEGF-A	Vascular Endothelial Growth Factor-A
