# Correction: Ficany et al. Epistaxis Prevention, Treatment, and Future Perspectives for Hereditary Hemorrhagic Telangiectasia. *J. Clin. Med.* 2025, *14*, 7724

**DOI:** 10.3390/jcm15062153

**Published:** 2026-03-12

**Authors:** Anthony Ficany, Marta Del Alamo, Carmelo Bernabeu, Claire L. Shovlin, Elisa Rossi

**Affiliations:** 1 INSERM, U1144 Optimisation Thérapeutique en Neuropharmacologie (OPTeN), Faculty of Pharmacy, Université Paris-Cité, 75006 Paris, France; anthony.ficany@u-paris.fr; 2 ECRIN–European Clinical Research Infrastructure Network, 75014 Paris, France; marta.delalamo@ecrin.org; 3 Department of Biomedicine, Centro de Investigaciones Biológicas “Margarita Salas”, Consejo Superior de Investigaciones Científicas (CSIC), 28040 Madrid, Spain; bernabeu.c@cib.csic.es; 4 National Heart and Lung Institute, Imperial College London, London W12 0NN, UK; c.shovlin@imperial.ac.uk; 5 Specialist Medicine, Imperial College Healthcare NHS Trust, London W12 0HS, UK

In the original publication [1] a sentence could have led to a misunderstanding or misinterpretation; therefore, we chose to correct it in **Section 2.4. Bevacizumab**, on page 14, as published. The sentence “Patients treated with bevacizumab showed non-statistically significant improvements in hematologic parameters and epistaxis” has been replaced by “Because the trial was underpowered, the primary outcome—the comparison of success rates defined as a ≥50% reduction in red blood cell (RBC) transfusions—did not reach statistical significance (63.6% vs. 33.3%). Nevertheless, the findings are considered clinically relevant, as the number of RBC transfusions decreased by 44% in the bevacizumab group compared with only 4% in the placebo group. Moreover, a statistically significant increase in hemoglobin levels was observed in the bevacizumab group relative to the placebo group”. The authors state that the scientific conclusions are unaffected. This correction was approved by the Academic Editor. The original publication has also been updated.
